# Inflow control can be safely used in laparoscopic subsegmentectomy of the liver: a single-center 10-year experience

**DOI:** 10.1186/s12893-023-02282-2

**Published:** 2023-12-06

**Authors:** Hao-Ping Wang, Teng-Yuan Hou, Wei-Feng Li, Chee-Chien Yong

**Affiliations:** Department of Surgery, Division of General Surgery, Chang Gung Memorial Hospital, Kaohsiung, No. 123, Dapi Rd., Niaosong Dist, Kaohsiung City, 833401 Taiwan

**Keywords:** Inflow control, Laparoscopic liver resection, Pringle maneuver

## Abstract

**Background:**

Several techniques have been developed to reduce blood loss in liver resection. The half-Pringle and Pringle maneuvers are commonly used for inflow control. This study compared the outcomes of different inflow control techniques in laparoscopic subsegmentectomy.

**Methods:**

From October 2010 to December 2020, a total of 362 laparoscopic liver resections were performed by a single surgeon (C.C. Yong) in our institute. We retrospectively enrolled 133 patients who underwent laparoscopic subsegmentectomy during the same period. Perioperative and long-term outcomes were analyzed.

**Results:**

The 133 patients were divided into 3 groups: no inflow control (n = 49), half-Pringle maneuver (n = 46), and Pringle maneuver (n = 38). A lower proportion of patients with cirrhosis were included in the half-Pringle maneuver group (*P* = .02). Fewer patients in the half-Pringle maneuver group had undergone previous abdominal (*P* = .01) or liver (*P* = .02) surgery. The no inflow control group had more patients with tumors located in the anterolateral segments (*P* = .001). The no inflow control group had a shorter operation time (*P* < .001) and less blood loss (*P* = .03). The need for blood transfusion, morbidity, and hospital days did not differ among the 3 groups. The overall survival did not significantly differ among the 3 groups (*P* = .89).

**Conclusions:**

The half-Pringle and Pringle maneuvers did not affect perioperative or long-term outcomes during laparoscopic subsegmentectomy. The inflow control maneuvers could be safely performed in laparoscopic subsegmentectomy.

## Introduction

Massive blood loss and blood transfusion during surgery adversely affect the outcomes of liver resection [1–5]. Thus, vascular inflow control is crucial in liver resection. Several techniques have been developed to reduce blood loss during liver resection, especially during laparoscopic surgery [6–8]. The technique of transient hepatic inflow occlusion for inflow control, also known as the Pringle maneuver, was first described by Pringle in patients with liver trauma and has been widely used in liver resection to reduce blood loss during liver resection surgery [9]. Moreover, this technique could be successfully performed during laparoscopic surgery [10]. However, the Pringle maneuver can lead to ischemic reperfusion injury [11, 12]. The effect of prolonged ischemic injury was more severe in patients with chronic liver diseases or cirrhosis [13–16]. Subsequently, hemihepatic vascular control, also known as the half-Pringle maneuver, was developed [17].

Compared with the Pringle maneuver, the half-Pringle maneuver can efficiently reduce blood loss and results in a lower risk of ischemic perfusion injury [18–21]. In addition, the half-Pringle maneuver prevents splanchnic congestion and has more favorable hemodynamic tolerability [22, 23]. Additional improvements in the technique and the nonrequirement of hilum dissection have enhanced the feasibility and safety of the half-Pringle maneuver technique [24–26].

Optimal inflow control relies on the location and extent of resection. This study compared the outcomes of different inflow control techniques in laparoscopic subsegmental hepatectomy.

## Materials and methods

**Fig. 1:**
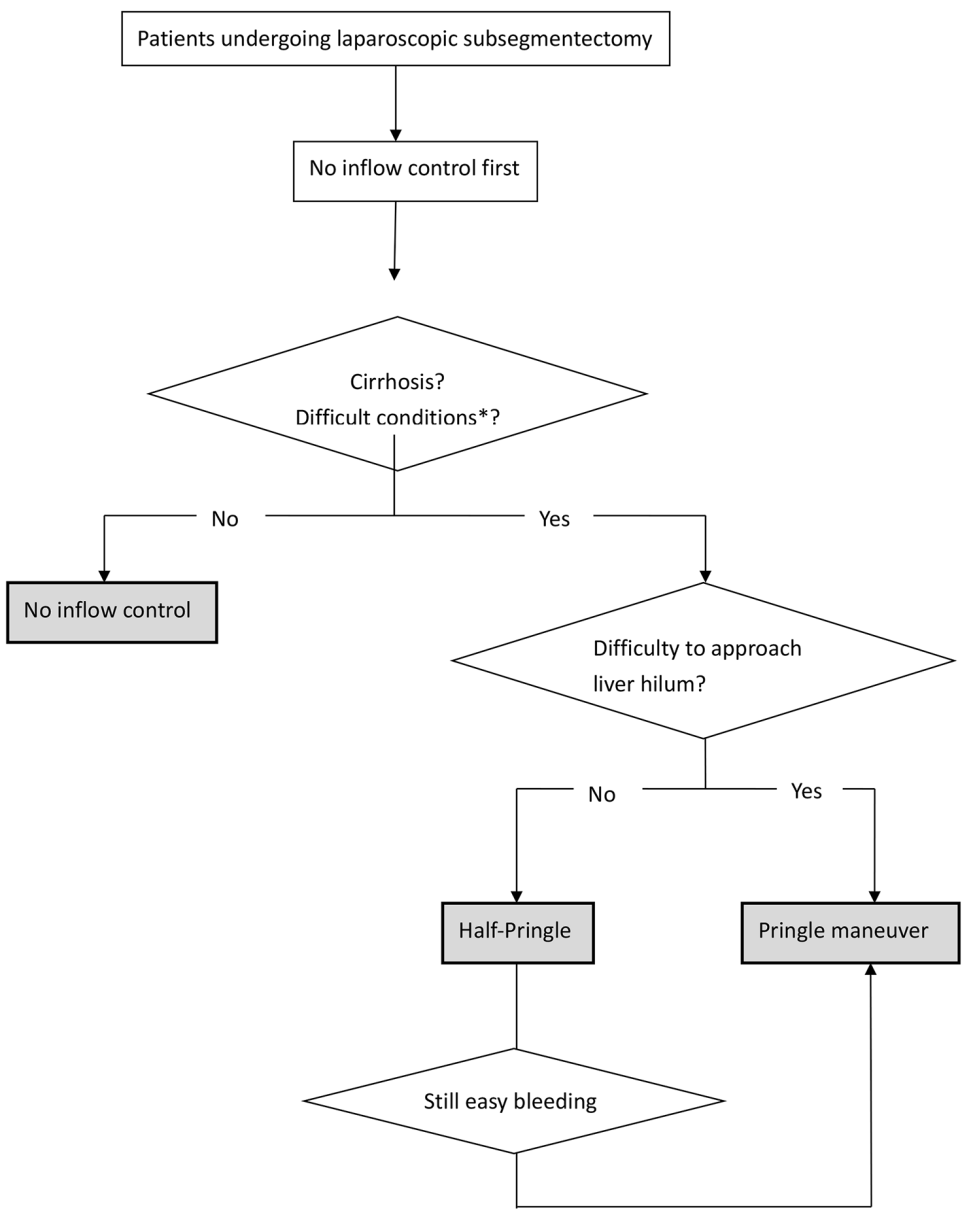
Algorithm of selection of inflow control methods in laparoscopic subsegmentectomy. * Difficult conditions meant easy bleeding or difficult tumor locations

This study was approved by the Chang Gung Medical Foundation Institutional Review Board (approval number: 202101465B0). We retrospectively reviewed the data of patients who underwent laparoscopic subsegmentectomy between October 2010 and December 2020 at Kaohsiung Chang Guang Memorial Hospital. We collected patient information on clinical characteristics and perioperative and long-term follow-up outcomes. During the study period, we performed laparoscopic hepatectomy in 362 patients, of whom 133 underwent laparoscopic subsegmentectomy for liver tumors, either malignant or benign lesions, that was conducted by a single surgeon (C.C. Yong). We retrospectively analyzed the 133 patients and divided them into 3 groups on the basis of the technique used for inflow control: no inflow control, half-Pringle maneuver, and Pringle maneuver. The surgeon decided which technique was used based on his own judgment and experience. Mostly, we would not use inflow control at first. In cirrhosis or difficult-to-approach cases (easy bleeding or difficult tumor locations), we would shift to the half-Pringle method as long as the liver hilum could be approached. If the case had adhesion over the liver hilum, we introduced the Pringle maneuver. (Fig. 1).

The liver anatomy and resection were defined according to the Brisbane 2000 terminology [27]. In all the patients included in this study, less than a segment of the liver was laparoscopically resected. The difficulty of liver resections was determined using the Iwate score [28] and Institut Mutualiste Montsouris (IMM) scoring system [29]. Surgical morbidities were examined using the Clavien–Dindo classification [30]. Cirrhosis was confirmed by pathologists with the specimen being resected.

### Surgical technique

The surgical technique was similar to that reported in previous studies [17, 31]. Briefly, the patient was placed in the supine position, and a 10-mm trocar was inserted in the umbilical wound for inflation. The pressure was maintained at 12 mmHg in the pneumoperitoneum. Other trocars were placed according to the procedure. Without hilar dissection, we placed a vascular clamp (Aesculap, Center Valley, PA, USA) at the right or left pedicle depending on the location of the lesion by adopting the extra-Glissonian approach to induce hemivascular occlusion. Subsequently, the half-Pringle maneuver was performed once the ischemic demarcation line was observed and the lesion was localized through intraoperative ultrasound. Initially, when the Pringle maneuver was needed, we used a large vascular clamp to occlude inflow. We have been using Huang’s loop since its development in 2018 [10]. Both the half-Pringle and Pringle maneuvers were intermittently performed with a 15-minute clamp and a 5-minute declamp.

Liver parenchymal transection was performed using the harmonic scalpel (Ethicon Endosurgery, Cincinnati, OH, USA) or the Thunderbeat (Olympus, Center Valley, PA, USA) with the Kelly clamp crushing technique. The vessels on the transection line were controlled using metal clips, vascular locks, or sutures.

### Peri-operative anesthesia setting

We routinely kept the CVP level below 5 mmHg before and during transecting the parenchyma. The ventilator setting depended on clinical condition. When bleeding was still noted after inflow was controlled, the bleeding was mainly from the venous back flow. Then the anesthesiologist would reduce the tidal volume down to 6 ~ 8ml/kg of ideal body weight to reduce blood loss.

### Postoperative care

All patients were transferred to the intensive care unit for further care postoperation. All care principles were the same in our center. Postoperative biochemical data, including serum total bilirubin, alanine aminotransferase, aspartate aminotransferase, albumin, creatinine, and C-reactive protein levels; prothrombin time; and international normalized ratio, were examined on postoperative days 1, 2, 3, 5, and 7. Posthepatectomy liver failure was defined and graded according to the International Study Group for Liver Surgery (ISGLS) guidelines [32]. Perioperative mortality was defined as death within 90 days of surgery and death during the same hospital admission for surgery.

### Statistical analysis

Categorical variables were compared using the Pearson chi-square test. All continuous variables are expressed as the means and were analyzed through one-way analysis of variance with post hoc Tukey’s test. The Kaplan–Meier curve was plotted for survival analysis, and data were compared among the 3 groups by using the log-rank test. A 2-sided *P* value of < 0.05 was considered statistically significant. Statistical analysis was performed using SPSS Statistics for Windows 20.0 (IBM Corp., Armonk, NY, USA).

## Results

**Fig. 2:**
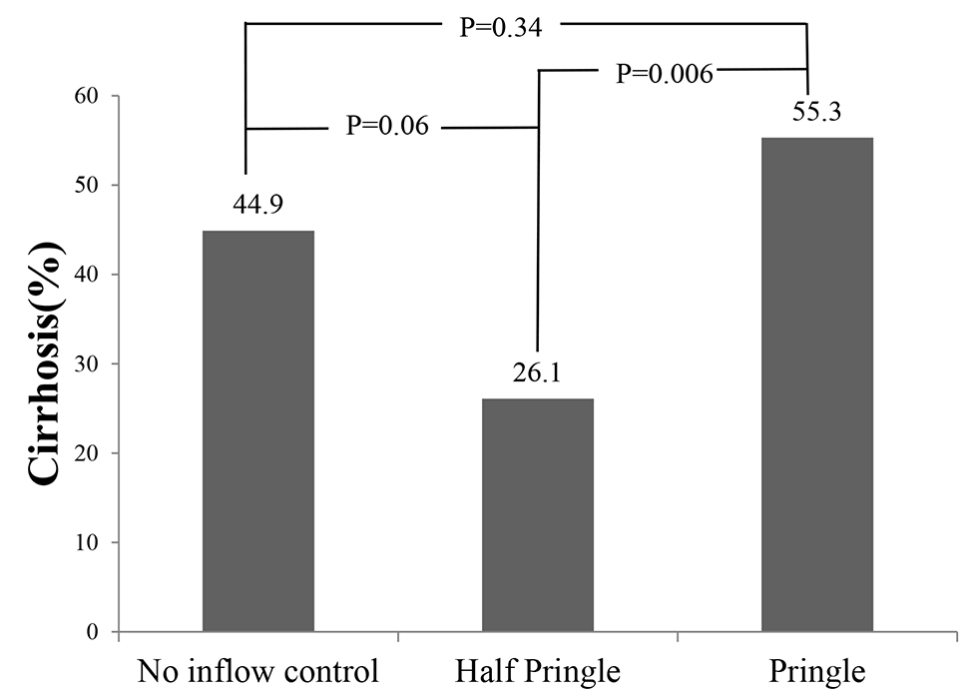
The proportion of patients with cirrhosis was lower in the half-Pringle group than in the other groups

From October 2010 to December 2020, a total of 133 patients who underwent laparoscopic subsegmentectomy were recruited for this study. All patients were divided into 3 groups on the basis of the technique used for inflow control during liver resection: no inflow control (n = 49), half-Pringle maneuver (n = 46), and Pringle maneuver (n = 38). Table 1 lists the demographics of the patients. The proportion of patients with cirrhosis was lower in the half-Pringle maneuver group than in the other groups (no inflow control vs. half-Pringle, *P* = .06; half-Pringle vs. Pringle, *P* = .006) (Table 1; Fig. 2). Furthermore, fewer patients in the half-Pringle maneuver group had previously undergone abdominal surgery (*P* = .01) and liver surgery (*P* = .02). A higher proportion of the patients in the no inflow control group exhibited tumors located at the anterolateral segments (Table 2, P = .001). Furthermore, the Iwate scores were lower in the no inflow control group (Table 2, P = .002). The IMM scores were lower in the no inflow control group (Table 2, P = .02).

**Table 1:** Clinical characteristics of patients (n = 133)

	No inflow control (n = 49)(%)	Half Pringle (n = 46)(%)	Pringle (n = 38)(%)	p value
Age	59.92	61.82	63.4	0.40
Sex(M:F)	29:20	32:14	24:14	0.57
BMI	25.45	24.59	24.95	0.53
HBV	25	25	19	0.91
HCV	11	12	13	0.46
Chirrhosis				0.02
No	27(55.1)	34(73.9)	17(44.7)	
Yes	22(44.9)	12(26.1)	21(55.3)	
Child-Pugh score				0.04
A	21(42.9)	10(21.7)	19(50.0)	
B	0	1(2.2)	0	
C	0	0	0	
Portal hypertension	22(44.9)	16(34.7)	16(42.1)	0.59
Previous abdominal operation	24(48.9)	9(19.6)	12(31.6)	0.01
Previous liver operation	10(20.4)	1(2.1)	6(15.8)	0.02
ASA score				0.40
0	0	0	0	
1	0	0	0	
2	27	21	15	
3	22	24	23	
4	0	1	0	

ASA: American Society of Anesthesiology, BMI: body mass index

**Table 2:** Tumor locations and difficulty scores

	No inflow control (n = 49)(%)	Half Pringle (n = 46)(%)	Pringle (n = 38)(%)	p value
Tumor location				0.001
Antero-lateral(S2-6)	47(95.9)	33(71.7)	25(65.8)	
Postero-lateral(S7-8)	2(4.1)	13(28.3)	10(26.3)	
Segment 1	0	0	3(7.9)	
Iwate tumor location score				0.002
1	6(12.2)	1(2.2)	3(7.9)	
2	32(65.3)	20(43.5)	10(26.3)	
3	9(18.4)	12(26.1)	12(31.6)	
4	0	1(2.2)	3(7.9)	
5	2(4.1)	12(26.1)	10(26.3)	
Iwate score				0.079
0	0	1(2.2)	0	
1	4(8.2)	1(2.2)	2(5.3)	
2	15(30.6)	11(23.9)	6(15.8)	
3	11(22.4)	14(30.4)	12(31.6)	
4	4(8.2)	2(4.3)	2(5.3)	
5	9(18.4)	11(23.9)	8(21.1)	
6	6(12.2)	5(10.9)	6(15.8)	
7	0	0	1(2.6)	
8	0	1(2.2)	1(2.6)	
Iwate difficult score				0.56
Low	30(61.2)	27(58.7)	20(52.6)	
Intermediate	19(38.8)	18(39.1)	16(42.1)	
Advance	0	1(2.2)	2(5.3%)	
IMM difficultScore				0.02
Low	34(69.4)	39(84.8)	31(81.6)	
Intermediate	15(30.6)	5(10.9)	4(10.5)	
High	0	2(4.3)	3(7.9)	

IMM: Institut Mutualiste Montsouris

The no inflow control group had a shorter operation time (Table 3, P < .001) and less blood loss (Table 3, P = .03). No differences in the need for blood transfusion, morbidity, or hospital days were noted among the 3 groups. The pathology results details were shown in Table 4.

**Table 3:** Perioperative outcomes, n = 133

	No inflow control (49)	Half pringle (46)	Pringle(38)	P value
Operative time(mins)	126.39	161.98	196.32	0.00
Clamp duration(mins)	0	37.3	42.47	0.00
eBlood loss(ml)	87.08*	168.37	208.42*	0.03
Blood transfusion	0(0)	2(4.3%)	4(10.5%)	0.06
Conversion	1(2.0%)	0(0)	3(7.9%)	0.10
Hospital days(days)	5.33	6.37	6.53	0.08
Morbidity(Clavien -Dindo				0.37
1	2	1	0	
2	1	4	1	
3a	0	1	1	
3b	1	0	0	
4a	1	0	0	
Complications > 3a	2(4.1%)	1(2.2%)	1(2.6%)	0.85
Mortality in 30 and 90 days	0	0	0	
Reoperation	1	0	0	0.42
Tumor size(mm)	24.78	24.87	28.06	0.30
Close margin(< 1 mm)	5	9	7	0.42

eBlood loss: estimated blood loss

*P = .03

**Table 4:** Pathology results

Pathology				0.07
Benign	11(22.4)	6(13.0)	2(5.3)	
FNH	2	3	0	
Hemangioma	2	1	1	
Regenerative nodular hyperplasia	1	0	0	
Inflammatory pseudotumor	4	1	0	
Focal fatty change	1	0	0	
Dysplasia	1	0	0	
IgG4 related disease	0	1	0	
Bile duct adenoma	0	0	1	
Malignancy	38(77.6)	40(87.0)	36(94.7)	
HCC	37	37	32*	
CholangioCA	1	1	4*	
CRLM	0	1	0	
Gallbladder cancer	0	0	1	
Breast cancer with liver metastases	0	0	1	
Malignant epithelioid hemangioendotheli-oma	0	1	0	

CholangioCA : cholangiocarcinoma, CRLM: colorectal liver metastases, FNH: focal nodular hyperplasia, HCC: hepatocellular carcinoma

* Two cases had combined hepatocellular carcinoma and cholangiocarcinoma

Four patients developed complications with a Clavien–Dindo classification higher than grade 3 (Table 5). A 71-year-old man in the half-Pringle maneuver group developed bile leakage postoperation. He underwent endoscopic retrograde biliary drainage with a plastic stent. However, the patient developed postendoscopic retrograde cholangiopancreatography pancreatitis after the procedure. A 58-year-old man in the no inflow control group developed ISGLS grade A posthepatectomy liver failure and pneumonia. Another 66-year-old man in the no inflow control group received surgery for bowel obstruction due to adhesion on postoperative day 7 and developed sepsis. A 64-year-old man in the Pringle maneuver group developed an intraabdominal abscess and was readmitted for CT-guided drainage on postoperative day 16. All patients recovered well after treatment and were discharged. No 30-day or 90-day mortality was noted in this study.

**Table 5:** Complications

Sex	Age	Viral hepatitis	ASA	Operation	Inflow control	Pathology	Tumor size (mm)	Cirrhosis	Operation time(mins)	Blood loss(ml)	Post operative stay(day)	Clavien-Dindo	Morbidity
Male	71	HCV	III	Laparoscopic S5 subsegmentectomy	Half-Pringle for 30 min	HCC	48	Nil	120	50	7	3 A	Bile leakage, ERCP relative pancreatitis
Male	58	HBV	III	Laparoscopic S2 subsegmentectomy	Nil	HCC	27	Yes	160	350	21	4 A	Post hepatectomy liver failure Pneumonia
Male	66	HBV	III	Laparoscopic S4b subsegmentectomy, left lower lung nodule VATS resection	Nil	HCC	22	Yes	120	150	14	3B	adhesion ileus status post adhesionlysis sepsis
Male	64	HBV	III	laparoscopic S5 and S8 subsegmentectomy,	Pringle for 69 min	HCC	25	Yes	240	200	8	3 A	Intra-abdominal abscesses, readmission for CT guided drainage

ASA: American Society of Anesthesiology, HCC: Hepatocellular carcinoma

**Fig. 3:**
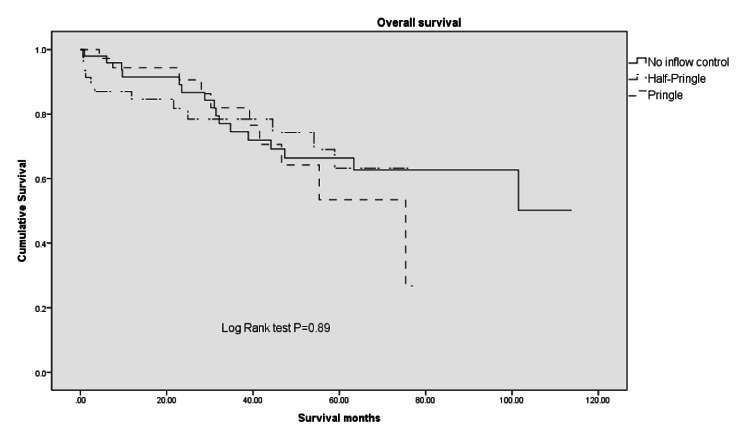
Overall survival. No significant difference in overall survival was noted among the three groups. Log Rank test: P = .89

No significant difference in overall survival was noted among the 3 groups (*P* = .89; Fig. 3).

## Discussion

Several inflow control techniques have been developed to reduce blood loss during liver resection, and the procedures have been reported to be feasible and safe [24–26]. We used both the Pringle maneuver and half-Pringle maneuver with the extra-Glissonian approach in laparoscopic subsegmentectomy. The results of our study revealed that the half-Pringle maneuver was performed in fewer patients who underwent previous abdominal or liver surgery because the adhesion of the hilum in previous surgery caused difficulty in performing the half-Pringle maneuver. In this situation, we performed the Pringle maneuver at a lower level of the portal triad to prevent hilum injury if inflow control was needed.

The choice of the optimal inflow control method relies on the complexity of procedures and the location of tumors. More definite inflow control might be required in more complicated conditions, such as in patients with portal hypertension. The half-Pringle maneuver might be appropriate in a small liver lesion in a few segments requiring minor resection [17, 31]. Our results indicated that a higher proportion of the patients with tumors located in the anterolateral segments (S2-S6) did not require any inflow control. Furthermore, more patients who received either the half-Pringle or Pringle maneuver had higher Iwate scores. This finding is consistent with our clinical judgment that the anterolateral location is easier to approach and reduces the need for inflow control. Higher Iwate scores indicated more difficulty in surgical techniques, thus possibly requiring inflow control. Although the Iwate score did not significantly differ among the groups, the IMM scores were lower in the no inflow control group. We believed that this result was not meaningful because our study focused only on subsegmentectomy; thus, selection bias might have been caused by other factors, such as tumor size and extent of liver resection, which did not differ among the groups. We recommend not using the Iwate or IMM score in laparoscopic subsegmentectomy as guidance for deciding the inflow control method.

In terms of perioperative data, the mean operation time was shorter in the no inflow control group, possibly because we did not perform inflow control in simple cases with less difficult location detection and less bleeding tendency and routinely used intermittent inflow control with either the half-Pringle or Pringle method rather than continuous occlusion. The declamp time might have caused an increase in operation time. Although the continuous half-Pringle method has been proven to be safe and beneficial compared with the intermittent Pringle maneuver even in patients with cirrhosis, [18, 19, 33, 34] we still used intermittent methods to reduce the risk of prolonged ischemia. For laparoscopic subsegmentectomy, the total operation time in our study group was approximately 200 minutes. The declamp time caused a slight increase in operation time. Thus, we believe that the benefits of intermittent inflow control would overcome the shortage of a prolonged operation time in limited liver resection.

The results of our study indicated that blood loss was lower in the no inflow control group. This might be a selection bias in this result. We attempted liver resection without any inflow control in easy-to-approach cases. However, we introduced inflow control once bleeding occurred during surgery and could not be controlled easily. Then, these cases were shifted to the inflow control groups. Thus, blood loss was greater in the half-Pringle and Pringle maneuver groups. Nevertheless, the postoperative data, including hospital days, major complications, and long-term survival, were not different among the 3 groups. Only one patient developed grade A posthepatectomy liver failure in the no inflow group. No patient developed liver failure in the half-Pringle or Pringle maneuver group.

The perioperative data and long-term outcomes were similar in the half-Pringle and Pringle maneuver groups; this finding is consistent with those of previous studies [35, 36]. Previous studies have reported fluctuations in liver enzymes post hepatectomy and less liver injury and earlier liver function recovery in patients who received the half-Pringle technique [18, 33, 37]. Our data revealed similar results for liver enzyme fluctuations, which are not shown in the tables. All the patients in the half-Pringle and Pringle maneuver groups recovered to baseline conditions without impaired perioperative and long-term outcomes; however, this finding was not clinically significant. The risk of posthepatectomy liver failure still cannot be neglected, especially in patients with chronic liver disease or cirrhosis. The choice of inflow control was dependent on the case. Similar to Horgan et al., [17] we suggest that the half-Pringle technique appears to be more suitable for patients with lesions in the hemiliver undergoing limited resection. The selective use of the half-Pringle technique can prevent unnecessary parenchymal ischemia. If the half-Pringle maneuver fails to occlude inflow, the Pringle maneuver might be an alternative effective technique. Although the Pringle maneuver might increase the risk of ischemic injury, no inferior short-term outcome of hepatectomy was seen in our study, even in the group with more cirrhotic liver and higher difficult scores. Therefore, although we need to adopt the Pringle maneuver in more challenging cases, laparoscopic hepatectomy can still be performed without compromising outcomes.

The half-Pringle technique resulted in adequate inflow control and was easy to perform. This technique avoids complex dissection to control selective inflow vessels. Previous studies [17, 31, 33] and the present study have demonstrated that this technique is feasible and safe for laparoscopic subsegmentectomy. Therefore, we suggest that the half-Pringle maneuver should be used when inflow control is needed. The half-Pringle maneuver not only reduces blood loss without requiring dissection of the hilum but also prevents unnecessary ischemic injury, which is a major concern in the Pringle maneuver.

This study has some limitations that should be addressed. First, this is a retrospective single-surgeon and single-center study; thus, selection bias was the biggest shortcoming. Second, our study was not randomized. The decision regarding the choice of the inflow control method was made by the surgeon. Finally, our data included different etiology cases, which might cause bias in long-term survival. A multicenter and multiple-surgeon study with a larger sample size should be conducted to confirm our results. Despite the limitations, we showed the solid data that inflow control did not compromise the perioperative and long-term outcomes in laparoscopic subsegmentectomy.

## Conclusions

The half-Pringle and Pringle maneuvers could be safely used when performed during laparoscopic subsegmentectomy without compromising perioperative or long-term outcomes. The half-Pringle technique is easy to perform and can be the first choice and alternative to the Pringle maneuver.

## Data Availability

The datasets used and/or analysed during the current study are available from the corresponding author on reasonable request.

## Abbreviations

IMM: Institut Mutualiste Montsouris
ISGLS: International Study Group for Liver Surgery
